# Enhancement of total sugar and lignin yields through dissolution of poplar wood by hot water and dilute acid flowthrough pretreatment

**DOI:** 10.1186/1754-6834-7-76

**Published:** 2014-05-23

**Authors:** Lishi Yan, Libing Zhang, Bin Yang

**Affiliations:** 1 Department of Biological Systems Engineering, Bioproducts, Sciences and Engineering Laboratory, Washington State University, Richland, WA 99354, USA

**Keywords:** Hot water, dilute acid, flowthrough pretreatment, Severity parameter, poplar wood, Enzymatic hydrolysis

## Abstract

**Background:**

Pretreatment is a vital but expensive step in biomass biofuel production. Overall, most of this past effort has been directed at maximizing sugar yields from hemicellulose and cellulose through trials with different chemicals, operating conditions, and equipment configurations. Flowthrough pretreatment provides a promising platform to dissolution of lignocellulosic biomass to generate high yields of fermentable sugars and lignin for biofuels productions.

**Results:**

Dissolution of xylan, lignin, and cellulose from poplar wood were significantly enhanced by water-only and dilute acid (0.05% w/w, H_2_SO_4_) flowthrough pretreatment when the temperature was raised from 200°C to 280°C over a range of flow rates 10-62.5 mL/min, resulting in more than 98% solid removal. Up to 40% of original xylan was converted to xylose in the hydrolyzate and the rest xylan was solubilized into xylooligomers with negligible furfural formation. Up to 100% cellulose was removed into hydrolyzate with the highest glucose yield of 60% and low 5-hydroxymethylfurfural (5-HMF) formation. The maximal recovered insoluble lignin and soluble lignin were 98% and 15% of original lignin, respectively. In addition, enzymatic hydrolysis of pretreated whole slurries was characterized under various enzyme loadings with or without Bovine serum albumin (BSA) treatment. More than 90% glucose yield and 95% xylose yield were obtained from enzymatic hydrolysis of dilute acid pretreated whole slurries with 10 mg protein Ctec 2 with 2 mg Htec2/g glucan + xylan.

**Conclusions:**

Nearly complete dissolution of whole biomass was realized through water-only and dilute acid flowthrough pretreatment under tested conditions. Temperature was considered as the most significant factor for cellulose degradation. The cellulose removal significantly increased as temperature reached 240°C for water-only and 220°C for dilute acid. Dilute acid pretreatment resulted in higher yields of recovered xylan and cellulose as monomeric sugars in the hydrolyzate than that for water-only pretreatment. Enzymes readily hydrolyzed the degraded cellulose and xylooligomers in pretreatment hydrolysate. Results suggested that kinetics controlled the flowthrough pretreatment of biomass dissolution, which was also affected by flow rate to certain extent.

## Background

Deconstruction of the naturally recalcitrant complex polymers comprising lignocellulosic biomass into simpler molecules that can be converted into useful fuels and chemicals is the major hurdle that needs to be overcome for economic viability [1]. Pretreatment is essential for achieving high yields of desirable products through overcoming the recalcitrance of lignocellulosic feedstocks, including: (1) hemicellulose, lignin and other compounds coating the surface of the cellulose microfibrils, and (2) the crystalline nature of the cellulose structure [2]. The feasibility of many pretreatment technologies has been proven at bench and pilot scales. However, a promising, less expensive path to improving the technology by the use of very dilute acid or even water-only technologies has been suggested [3]. Apart from their economic viability, these technologies have several powerful attributes including high yields, high cellulose digestibility, low chemical usage, and fewer safety and environmental concerns [4]. Unfortunately, these alternative approaches are typically difficult to implement due to the high water consumption [2].

A number of studies over the years have shown that passing liquid hot water with and/or without addition of chemicals (for example, acid, alkali) [5-9] through cellulosic biomass at high temperatures produces highly digestible cellulose, high yields of sugars from hemicelluloses [8,10-12], over 85% lignin removal [13], and liquid hydrolyzate that appears more compatible with fermentative organisms [14]. Increasing the temperature of hot water flowthrough pretreatments to 225 to 270°C within or above saturated steam pressure also solubilizes the cellulose [10,15]. For example, as early as the 1970s and 1980s, Bobleter and his colleagues [16] applied hot water flowthrough process to hydrolyze air-dried pure cellulose at 260 to 270°C. Up to 52% glucose yield and 10% 5-hydroxymethylfufural (5-HMF) were obtained through hydrolyzing cellulose under 265°C at a flow rate of 12 mL/minute. Furthermore, employing a two-stage (230°C for 15 minutes and 270°C for 15 minutes) semi-flow hot water pretreatment at a flow rate of 10 mL/minute under pressure of 10 Mpa was found to remove 100% xylan, 89.4% lignin and 79.5% cellulose, respectively. However, substantial sugar degradations, including furfural (approximately 6.9%), 5-HMF (approximately 6.9%), glycoaldehyde (approximately 2.7%), were observed [17]. Results from flowthrough pretreatment at elevated temperatures provide invaluable evidence of the deconstruction pattern of biomass and improve understanding of how releases of various biomass fractions are related while providing new fundamental insights into hydrolysis kinetics that are not possible to observe in batch operations.

Enzymatic hydrolysis of pretreated whole slurry, including hydrolyzate and pretreated solid residues, in a simplified single step that could lead to lower capital and operating costs [18], depending on the technologies and conditions applied, was shown to be challenging. The nature of both pretreatment hydrolysate and pretreated residues strongly affect the digestibility of pretreated whole slurries [19,20]. Bovine serum albumin (BSA) treatment with the mechanism attributed to promoting blocking enzymes from non-productive binding [20], stabilizing enzyme [21], and detoxification of hydrolyzate [22] was shown promising in improving the efficacy of enzymatic hydrolysis. Therefore, BSA treatment coupled with advanced pretreatment method has the potential to realize enzymatic hydrolysis of pretreated whole slurry with high yield.

In this study, poplar wood was pretreated in a flowthrough system at elevated temperatures of 200 to 280°C under varied conditions (0 to 30 minutes, H_2_SO_4_ 0.0 to 0.05% (w/w), and flow rates of 10 to 62.5 mL/minute) to investigate effects on yields of total mass, lignin and sugars (mono and oligomer), as well as subsequent enzymatic hydrolysis of pretreated whole slurries. In addition, the evaluation of subsequent enzymatic hydrolysis of pretreated whole slurries at varied enzyme loadings, with and without BSA was compared. We seek to understand reasons for differences in the performance, and establish knowledge gained to help apply and improve pretreatment technology.

## Results and discussion

### Effects of preheating on removal of total mass, xylan, lignin, and cellulose

At the start of reaction, the temperature transients that occur as the reactor is heated from ambient to reaction temperature must be considered [23]. A series of experiments for water-only (220 to 280°C) and 0.05% (w/w) H_2_SO_4_ operations (200 to 250°C) with a flow rate of 10.0 to 62.5 mL/minute were carried out to determine the poplar wood degradation performance during the preheating process (provided in Additional file 1: Figure S1 and Additional file 2: Table S1). As presented in Figure S1, preheating time from room temperature to 200 to 280°C ranged from 1.2 to 2.8 minutes. Table S1 shows that more than 76% xylan and 52% lignin were removed during preheating to 220°C for water-only pretreatment. Elevating target temperature or adding acid increased the removal of both xylan and lignin. Overall, up to 100% of xylan, 49% cellulose and 87% lignin were removed into the hydrolyzate through the preheating processes under tested conditions. Most of the dissolved xylan and cellulose for these preheating processes was in the form of oligomers with a small amount of xylose and glucose, and negligible degradation compounds.

### Effects of pretreatment severity parameter on removal of total mass, xylan, lignin, and cellulose

**Figure 1:**
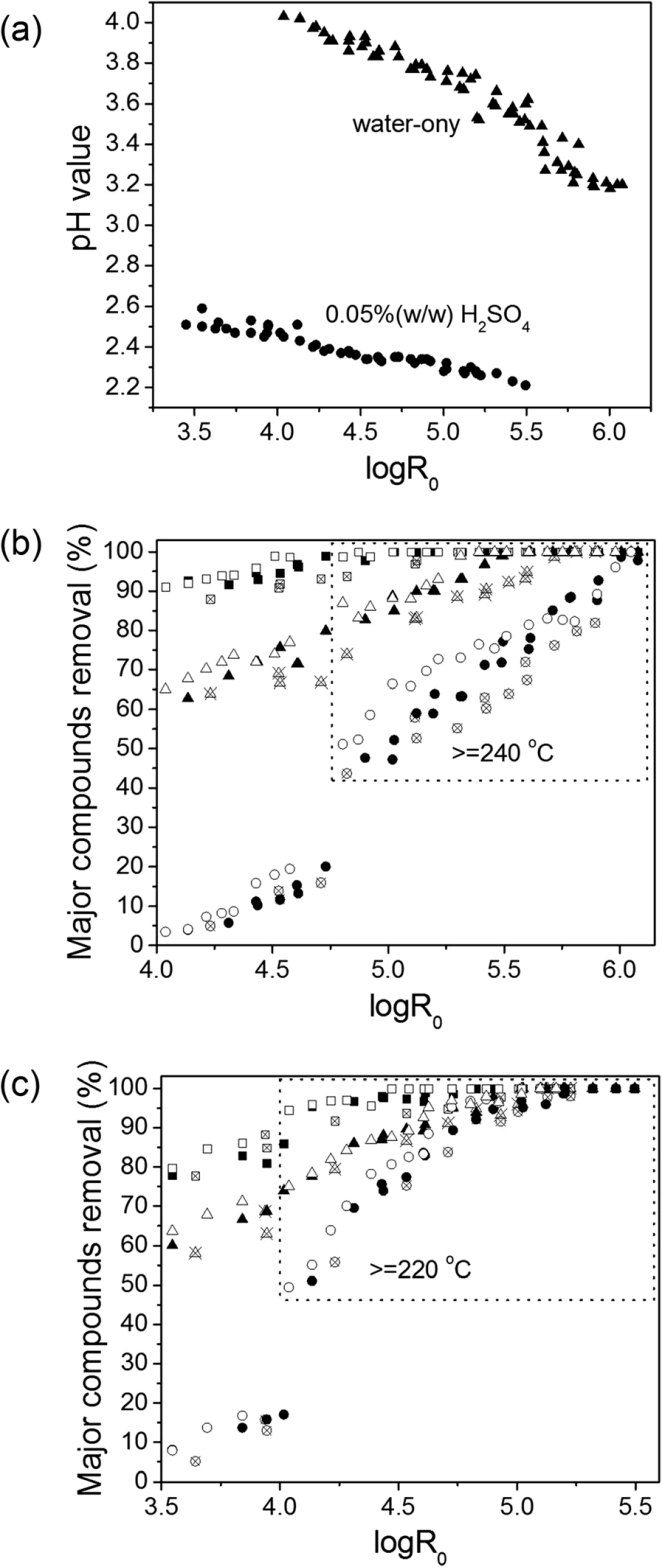
**Effect of severity parameter (logR**_**0**_**) on the removal of xylan, lignin and cellulose. (a)** Log R_0_ versus pH: solid triangles = water-only pretreatment; solid circles = 0.05% (w/w) H_2_SO_4_ pretreatment. **(b)** Log R_0_ versus removal of xylan, lignin and cellulose with water-only pretreatment. **(c)** Log R_0_ versus removal of xylan, lignin and cellulose with 0.05% (w/w) H_2_SO_4_. In **(b)** and **(c)**: crossed squares = xylan removal (10 mL/minute), solid squares = xylan removal (25 mL/minute); open squares = xylan removal (62.5 mL/minute); crossed triangle = lignin removal (10 mL/minute), solid triangle = lignin removal (25 mL/minute), open triangle = lignin removal (62.5 mL/minute); crossed circle = cellulose removal (10 mL/minute); solid circle = cellulose removal (25 mL/minute); open circle = cellulose removal (62.5 mL/minute).

Removal of xylan, lignin, and cellulose from poplar wood through flowthrough pretreatment under target temperatures ranging from 220 to 280°C for water-only, and 200 to 250°C for dilute acid pretreatment, for 0 to 30 minutes (including preheating time), and at flow rates of 10 mL/minute, 25 mL/minute and 62.5 mL/minute, were investigated. The pH of each liquid sample was promptly measured with a pH meter upon cooling to room temperature. Water-only pretreated hydrolyzates with a pH value of 4.0 to 3.2 and dilute acid pretreated hydrolyzates with a pH value of 2.6 to 2.2 were observed at corresponding severities (Figure 1a).

#### Xylan removal

It is known that hemicellulose and lignin are covalently linked in biomass, and the high solubility of hemicellulose oligomers can facilitate their dissolution, thus these soluble compounds can be removed before any further reactions occur [24]. Figure 1b and c show that increasing severities for water-only and dilute acid pretreatment enhanced xylan removal. Almost all xylan was removed when reaction severity logR_0_ > 4.5 and logR_0_ > 4.2 for water-only and dilute acid operations, respectively. As expected, the most readily hydrolyzed constitute, such as xylan, is partially deacetylated as well as depolymerized in the presence of acidic water [7]. The sulfuric acid addition increased the rate of xylan removal for flowthrough systems (Figure 1c). However, water-only flowthrough pretreatment at lower temperatures (160 to 220°C) led to almost total xylan removal at analogous severities (log R_0_ > 4.5) [13], suggesting that temperature had limited effect on xylan removal. On the other hand, increasing the flow rate from 10 mL/minute to 62.5 mL/minute appeared to have limited effects on xylan solubilization (less than 10% xylan removal increased under experimental conditions).

#### Lignin removal

The apparent coupling of lignin and hemicellulose release during flowthrough pretreatment suggests that hemicelluloses-lignin oligomers dissolution rates and solubility limitations play key roles in realizing high lignin removal [24]. Results (Figure 1b and c) showed that increasing pretreatment severity improved lignin removal for both water-only and dilute acid pretreatment although less portion of lignin than xylan was removed at a similar severity. At the lowest severity tested, about 65% and 60% lignin was removed by water-only and dilute acid, respectively. For water-only pretreatment, about 85% lignin removal with a flow rate of 10 ml/minute and nearly 100% lignin removal with a flow rate of 62.5 ml/minute at logR_0_ = 5.3 were obtained, while for dilute acid pretreatment, nearly 90% lignin removal with a flow rate of 10 mL/minute and 95% lignin removal with a flow rate of 25 mL/minute at logR_0_ = 4.7 were observed. At all comparable values of pretreatment severity, higher flow rate resulted in larger portions of lignin being removed by water-only pretreatment. Results suggested that increasing flow rate from 10 mL/minute to 62.5 mL/minute could improve lignin removal by 5 to 15% for water-only and around 5% for dilute acid, respectively.

#### Cellulose removal

Unlike xylan and lignin, cellulose consists of cellulose Iβ and Iα, which are both held together via a network of hydrogen hydrophobic interactions, causing deconstruction of the crystals challenging [25]. Thus, the removal of cellulose was only 5 to 20% at logR_0_ = 4.0 to 4.7 with flow rates ranging from 10 to 62.5 mL/minute for water-only pretreatment, which increased gradually as severity was elevated (Figure 1b). Interestingly, cellulose removal rapidly increased to 40% at 10 mL/minute and 50% with 62.5 mL/minute flow rate when severity logR_0_ reached 4.8 at 240°C. As previously reported, cellulose Iβ underwent a transition into an amorphous structure when temperature increased to around 220 to 230°C [26]. Increasing the temperature of hot water and/or dilute acid flowthrough pretreatments to 220 to 270°C within or above saturated steam pressure solubilizes the cellulose [10,15]. As severity further increased to 6.0 with a temperature ranging from 240 to 270°C, cellulose removal was continuously improved until nearly 100% removal was reached with water-only. The removal of almost all cellulose also corresponded to more than 98% total biomass dissolution. For dilute acid pretreatment, abrupt enhancement of cellulose removal from 16% to about 50% was also observed but at lower severity of 4.0 and lower temperature of 220°C. When logR_0_ was higher than 4.0 at a temperature above 220°C, cellulose removal was rapidly improved to nearly 100% at logR_0_ = 5.0. Previous water-only or dilute acid flowthrough pretreatment studies also revealed that small amount of cellulose was hydrolyzed at lower temperatures of 180 to 220°C [27], whereas cellulose decomposed significantly at higher temperatures (that is, >250°C) [5,10]. Results showed that among tested factors (for example, acid concentration, time, etcetera), temperature could play an important role in explaining the effects of acidic aqueous pretreatment on cellulose dissolution. It was reported that a sudden departure of the cellulose degradation rate constants from a normal Arrhenius pattern occurs around 215°C with 0.07% (w/w) H_2_SO_4_[28].

In addition, flow rate appeared to affect cellulose removal to some extent. For example, increasing the flow rate from 10 mL/minute to 62.5 mL/minute for water-only pretreatment resulted in 3% to 15% higher cellulose removal at comparable severities (Figure 1b).

### Sugars, sugar degradation products and lignin recovery through flowthrough pretreatment

#### Xylan recovery

**Figure 2:**
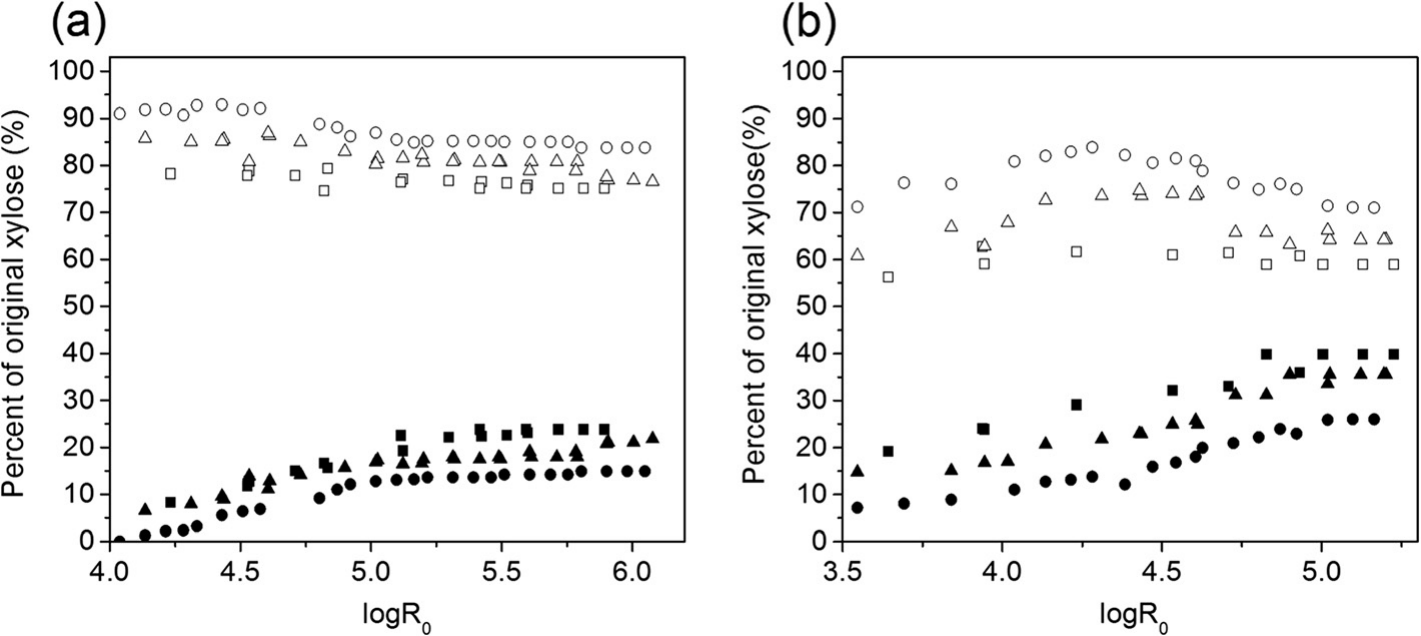
**Effect of logR**_**0 **_**on xylan recovery with (a) water-only and (b) 0.05% (w/w) H**_**2**_**SO**_**4**_**.** Solid squares = xylose (10 mL/minute); open squares = xylooligomers (10 mL/minute); solid triangles = xylose (25 mL/minute); open triangles = xylooligomers (25 mL/minute); solid circles = xylose (62.5 mL/minute); open circles = xylooligomers (62.5 mL/minute).

Figure 2a presents xylose and xylooligomers yield from poplar wood by water-only flowthrough pretreatment. Results showed that xylooligomers were predominantly recovered xylan in filtered pretreatment hydrolyzate at all tested severities. Higher than 75% xylooligomer yields were observed while xylose yields were less than 25%.

Previous studies reported that recovered xylan pretreated at lower temperatures (200°C) is also primarily composed of xylooligomers with even less xylose (<10% xylose yield) [24]. Xylooligomer yields decreased slightly as logR_0_ increased while the corresponding xylose yield increased. It indicated that the increased severity could shift the distribution of generated sugars to monomers. Conversely, increasing the flow rate from 10 mL/minute to 62.5 mL/minute resulted in 10 to 20% improvement of xylooligomer yield while the corresponding xylose yield decreased by about 15%.

Figure 2b revealed that 56.2 to 71.2% xylooligomer yield and 7.2 to 19.2% xylose yield was obtained at around logR_0_ = 3.5 with flow rates ranging from 10 mL/minute to 62.5 mL/minute for dilute acid pretreatment. As severity increased, xylose yield gradually increased to 39.9, 35.6 and 26.0% at logR_0_ = 4.9 with a flow rate of 10 mL/minute, 25 mL/minute and 62.5 mL/minute, respectively, then remained similar value when severity was between 5.0 and 5.5. On the contrary, xylooligomer yield climbed to the peak yields of 74.7 and 83.9% around logR_0_ = 4.3 to 4.4 with a flow rate of 25 mL/minute and 62.5 mL/minute, respectively, then it gradually declined as severity increased further. Compared to water-only pretreatment, adding dilute acid increased the xylose yield (Figure 2b). For example, at a flow rate of 25 mL/minute, the xylose yield was observed 14.7 to 35.6% at logR_0_ = 3.5 to 5.5, much higher than 6.6 to 21.8% xylose yield obtained with water-only at similar severity parameters. On the contrary, the xylooligomer yield with dilute acid decreased to 60.8 to 74.7% compared with 76.6 to 87.0% for water-only. In addition, results showed that with flow rate of 10 mL/minute, logR_0_ = 5.9 was necessary to reach the highest xylose yield of 25% for water-only while dilute acid pretreatment yielded similar xylose at logR_0_ = 3.9. It appeared that lower severity value was required to reach similar xylose yield for dilute acid than water-only flowthrough pretreatment.

Results indicated that flow rate had more significant effects on xylose and xylooligomer yield for dilute acid: 10 to 20% increase of xylooligomer yield and 12 to 20% decline in xylose yield when flow rate was increased from 10 mL/minute to 62.5 mL/minute. In addition, results indicated that almost all the removed xylan was recovered as xylose and xylooligomers with negligible amount of degradation compounds. For example, almost 100% xylan removal resulted in 98.2 and 98.8% xylose plus xylooligomers yields for water-only (logR_0_ = 5.0, 25 mL/minute) and dilute acid (logR_0_ = 4.8, 25 mL/minute), respectively. As in the above discussion of effects of preheating on removal of total mass, xylan, lignin, and cellulose, most of soluble xylooligomers were swept out of the reactor before any further reactions occurred in the preheating procedure, during which the temperature was lower than the target temperature, thus led to low formation of furfural [2].

#### Cellulose recovery

**Figure 3:**
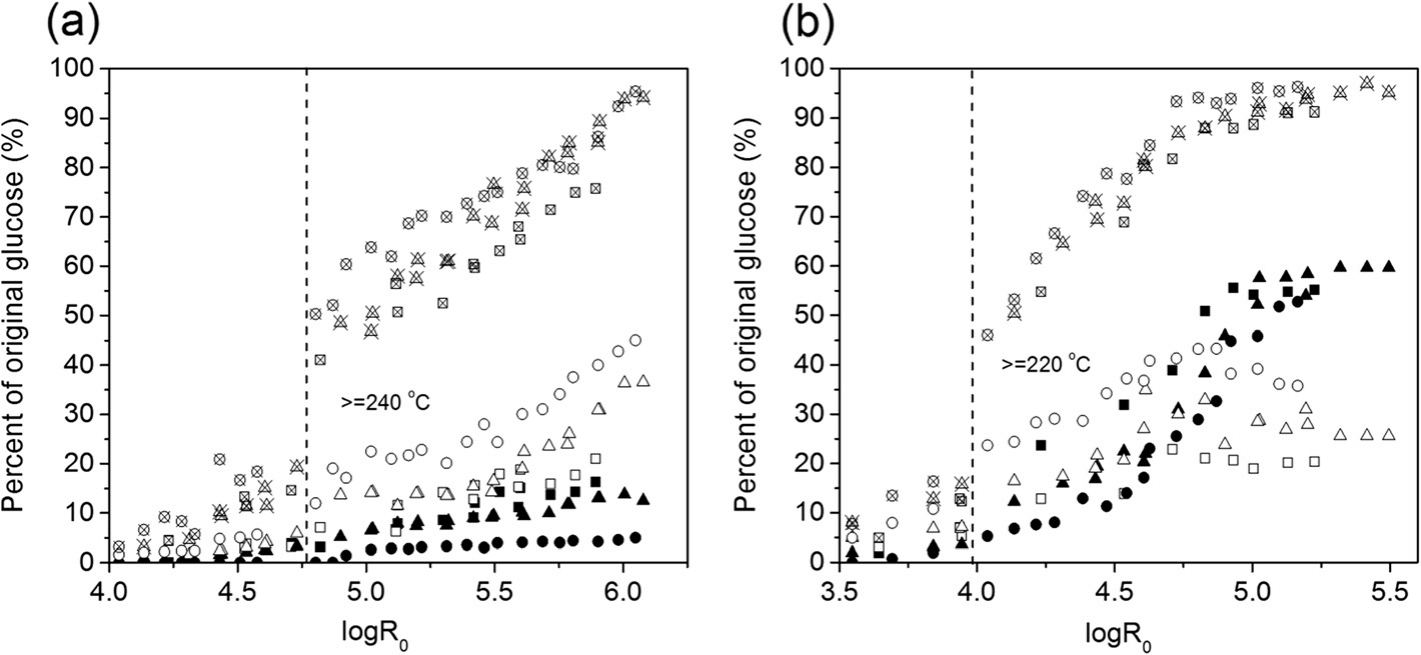
**Effect of logR**_**0 **_**on cellulose recovery by (a) water-only and (b) 0.05% (w/w) H**_**2**_**SO**_**4 **_**pretreatment.** Solid squares = glucose (10 mL/minute); open squares = glucose oligomers (10 mL/minute); crossed squares = total glucan recovery (10 mL/minute); solid triangles = glucose (25 mL/minute); open triangles = glucose oligomers (25 mL/minute); crossed triangles = total glucan recovery (25 mL/minute); solid circles = glucose (62.5 mL/minute); open circles = glucose oligomers (62.5 mL/minute); crossed circles = total glucan recovery (62.5 mL/minute).

Both water and dilute acid pretreatments at elevated temperature with increased pretreatment severity (for example, temperature, acid concentration, and reaction time) lead to the decrystallization of cellulose structure and further release of glucose by cleavage of β-1,4-glycosidic bonds hence promote the hydrolysis of cellulosic biomass [10,17,29]. Yields of glucose and soluble glucose oligomers in filtered pretreated hydrolysate can indicate the yields of soluble cellulosic fractions, while the total glucan recovery after enzymatic hydrolysis of unfiltered hydrolyzate can reveal the total glucan available in pretreated hydrolyzate. In this study, yields of glucose and soluble glucose oligomers in filtered pretreated hydrolysate and the total glucan recovery after enzymatic hydrolysis of unfiltered hydrolyzate were compared. Figure 3a shows that the yields of glucose and soluble glucose oligomers in filtered hydrolyzate and total glucan recovery increased as severity was elevated for water-only pretreatment. Results showed that both glucose and glucose oligomer yields increased gradually as severity increased from 4.0 to 6.0 for all tested flow rates except for glucose oligomer yield, which showed a slightly abrupt increase around logR_0_ = 4.8 for a flow rate of 25 mL/minute and 62.5 mL/minute. The highest glucose yield of 16.2% was achieved at a high severity around logR_0_ = 5.8 with a flow rate of 10 mL/minute while the highest glucose oligomer yield of 45.0% was found at logR_0_ = 6.0 with a flow rate of 62.5 mL/minute. Correspondingly, although the total glucan recovery increased gradually with log R_0_ < 4.8, an abrupt increase was observed at about logR_0_ = 4.8 and it continuously rose rapidly to around 95% at logR_0_ ranging from 4.8 to 6.0 as temperature was higher than 240°C. These results indicated that the total glucan recovery was comparable to that of cellulose removal (Figure 1b). Furthermore, it was noteworthy that the difference between the total glucan recovery and the sum of glucose and glucose oligomer yields, which implied the yield of removed insoluble cellulosic fractions, also showed abrupt enhancement when logR_0_ was around 4.8 and temperature was higher than 240°C. At logR_0_ = 6.0, nearly 100% cellulose removal merely resulted in 50% glucose plus glucose oligomer yield and 1.6% 5-HMF yield (see Table 1) while the total glucan recovery was about 95% with a flow rate of 62.5 mL/minute. It indicated that when logR_0_ was >4.8, besides glucose and glucose oligomers and the small amount of cellulose in pretreated solid residues, the remainder cellulosic fractions in hydrolyzate was predominately in the form of insoluble cellulosic fractions.

**Table 1:** Effects of reaction parameters on the generation of 5-HMF and furfural during flowthrough pretreatment

**Flow rate (mL/minute)**	**Temperature (°C)**	**Reaction time (minutes)**^**a**^	**H**_**2**_**SO**_**4 **_**concentration (%)**	**pH**	**5-HMF (%)**	**Furfural (%)**
10	220	5	0	3.98	0	0
10	220	10	0	3.90	0	0
10	240	5	0	3.77	1.8	0
10	240	10	0	3.67	3.1	0
10	250	5	0	3.66	2.8	0.7
10	250	10	0	3.56	3.1	0.8
10	220	5	0.05	2.41	1.3	0
10	240	5	0.05	2.36	4.5	1.2
25	240	6	0	3.73	0	0
25	240	10	0	3.67	0	0
25	270	6	0	3.21	0	0
25	270	10	0	3.18	0	0
25	280	6	0	3.19	3.0	0.5
25	240	6	0.05	2.34	0	0
25	250	6	0.05	2.28	2.6	0.3
62.5	270	5.6	0	3.23	0	0
62.5	280	5.6	0	3.12	1.6	0
62.5	250	5.6	0.05	2.33	1.2	0

^a^Including the preheating time.

Yields of glucose and soluble glucose oligomers in pretreatment hydrolyzate increased more rapidly with dilute acid than those with water only at similar severity parameters (Figure 3b). Results suggested that the addition of acid accelerated the hydrolysis rate of cellulose to glucose oligomers, and subsequently to glucose. Glucose yield increased gradually with severity at tested flow rates, then climbed steeply to the maximum yield of 59.6% with a flow rate of 25 mL/minute at logR_0_ = 4.1 to 5.5, while soluble glucose oligomer yield continuously increased to the peak yield of 43.3% at logR_0_ = 4.8 with a flow rate of 62.5 mL/minute, then declined with all tested flow rates as severity further increased. Within the range of tested severity parameters and flow rates, it was found that the maximum yield of glucose plus soluble glucose oligomers by dilute acid pretreatment reached 86.3%, much higher than that of 50.2% for water-only operation. In addition, glucose yield by dilute acid was much higher than that by water-only pretreatment. For example, with dilute acid pretreatment, 12.3 to 59.6% glucose yield was obtained at logR_0_ = 4.1 to 5.5 with a flow rate of 25 mL/minute. In comparison, under similar conditions (that is, temperature, time, flow rate), glucose yield reached 0.0 to 9.5% for water-only pretreatment. The total glucan recovery pretreated with dilute acid also increased as severity increased and showed abrupt enhancement at lower severity log R_0_ = 4.0 and a lower temperature of 220°C than water-only pretreatment. At logR_0_ = 5.5 with a 25 ml/minute flow rate with dilute acid, where 100% cellulose was removed, 84.5% glucose plus soluble glucose oligomer yield with negligible 5-HMF was observed and 98.7% original glucan was recovered in pretreatment hydrolyzate. This indicated around 14.2% insoluble cellulosic fractions were formed.

Results showed that soluble glucose oligomer yields increased with flow rate for water-only and dilute acid pretreatment (Figure 3a and b). This could be explained by more glucose oligomers dissolving at higher flow rates due to the presence of a greater amount of water. Meanwhile, the faster flow could also rapidly remove dissolved oligomers from the reactor before they further hydrolyze into monomers. On the other hand, a lower flow rate increased the portion of glucose in pretreatment hydrolyzate. For example, at logR_0_ = 5.9, when the flow rate decreased from 62.5 mL/minute to 10 mL/minute, the glucose yield increased from 4.2 to 16.3%, while the glucose oligomer yield declined from 40.1 to 21.0% with water-only. The total glucan yield increased 10 to 20% and 5 to 10% for water-only and dilute acid, respectively, when the flow rate was increased from 10 mL/minute to 62.5 mL/minute. Thus, flow rate appeared to influence the generation of glucose and glucose oligomers in a manner similar to its effect on the yields of xylose and xylooligomers.

#### Sugar degradation patterns

Biomass-derived monomeric sugars can be further dehydrated into furans (furfural and 5-HMF) [30,31], which in turn can degrade into organic acids, such as levulinic acid [32], resulting in reduced fermentable sugar yield. As shown in Table 1, at a flow rate of 10 mL/minute, 3.1% 5-HMF yield was observed at 240°C after 10 minutes with water only, whereas elevating the flow rate to 25 mL/minute resulted in negligible 5-HMF yield. Even when the temperature was raised to 270°C, 5-HMF yield remained negligible with a flow rate of 25 mL/minute: 0.7% furfural was formed under 250°C at 10 minutes when employing a flow rate of 10 mL/minute. However, furfural became imperceptible when the flow rate was raised to 25 mL/minute and 62.5 mL/minute under identical or higher temperatures (for example, 270°C). Results indicated that higher flow rates of 25 mL/minute and 62.5 mL/minute led to both negligible amounts of 5-HMF and furfural at elevated temperatures for both water-only (≤270°C) and 0.05% (w/w) H_2_SO_4_ (≤240°C) operations. Compared to other studies conducted at analogous temperatures (265 to 270°C, water only) with lower flow rates (10 to 12 mL/minute), higher amounts of 5-HMF (approximately 10%) and furfural (approximately 6.9%) were observed [16,17]. A flow rate of 25 mL/minute with relatively lower water consumption appeared to be desired for higher sugar concentration. Results suggested that undesirable decomposition reactions of glucose and xylose to 5-HMF and furfural can be limited by controlling severity parameter and flow rate. In line with this reasoning, it is interesting to note that the yields of 5-HMF and furfural observed under water-only and dilute acid operations under analogous severities were comparable. The yields of furfural were lower than those of 5-HMF under these tested conditions although xylose was much easier to degrade than glucose [33]. The possible explanation was that a much higher fraction of xylan was swept out of a reactor in the preheating period due to a greater solubility when temperature and flow rate increased, and acid was added.

#### Lignin recovery

**Figure 4:**
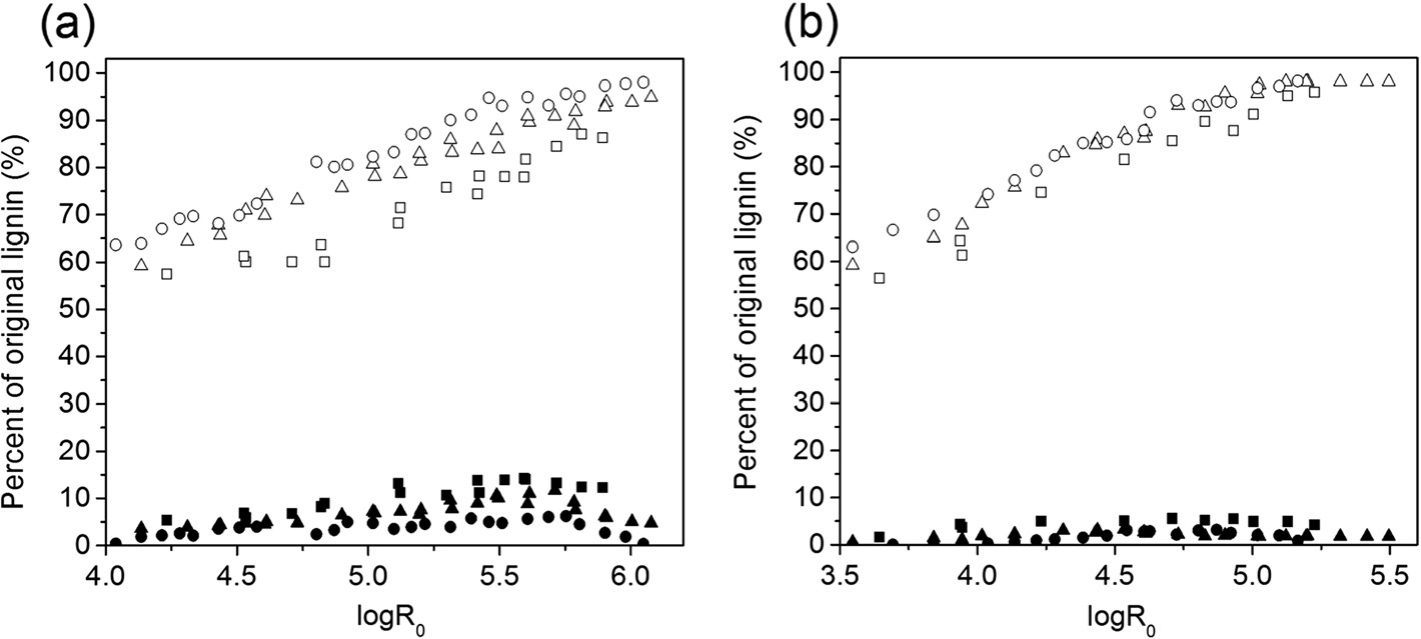
**Effect of logR**_**0 **_**on lignin recovery under (a) water-only and (b) 0.05% (w/w) H**_**2**_**SO**_**4 **_**conditions.** Solid squares = soluble lignin (10 mL/minute); open squares = insoluble lignin (10 mL/minute); solid triangles = soluble lignin (25 mL/minute); open triangles = insoluble lignin (25 mL/minute); solid circles = soluble lignin (62.5 mL/minute); open circles = insoluble lignin (62.5 mL/minute).

Lignin is believed to depolymerize and micellarize under acidic conditions via both homolytic and acidolytic cleavage into low molecular-weight lignin globules [34-36]. As acidic water passes through the material, especially at high flow rates, highly reactive nucleophilic carbonium ion intermediates are formed within the lignin structure, and can react further leading to the cleavage of predominant β-O-4 bonds. This realizes efficient depolymerization of lignin, which can be quickly and continuously swept out of the reactor to limit the simultaneous repolymerization reaction and re-precipitation of the deplomerized lignin at ambient temperature [35,36]. As shown in Figure 4, a large fraction of the recovered lignin in the hydrolyzate during flowthrough reactions was in the insolubilized form for both water only and dilute acid. For example, insoluble lignin recovery ranged from 59.3 to 87.8% under water-only conditions (25 mL/minute) when the severity was increased from logR_0_ = 4.1 to logR_0_ = 5.5. In contrast, adding acid significantly enhanced the insoluble lignin recovery from about 75.6 to 98.0% when logR_0_ increased from 4.1 to 5.5. Apart from insoluble lignin, a small fraction of removed lignin was solubilized in the hydrolyzates for both water-only and dilute acid flowthrough pretreatment. For water only, the yield of soluble lignin was 3.6% at logR_0_ = 4.1, then increased slowly as the severity parameter increased. The highest yield was 11.7% at logR_0_ = 5.7, which then decreased when logR_0_ was continuously increased. By comparison, soluble lignin recovery for dilute acid pretreatment was much less than that with water only at all severity ranges. Adding acid resulted in the maximal soluble lignin yield of 5.6% at logR_0_ = 4.7. Flow rate effected the distribution of the removed lignin to some extent and it was more apparent for water-only pretreatment. Although a higher flow rate (for example, 62.5 mL/minute) resulted in more lignin removal than a lower flow rate (for example, 10 mL/minute), it was found that 3 to 9% increase in soluble lignin yield was realized when the flow rate declined from 62.5 mL/minute to 10 mL/minute for water only. It was plausible that a lower flow rate increased the exposure of removed lignin under high temperature for decomposing into low molecular weight compounds.

The gas chromatography/mass spectrometry (GC/MS) analysis was essentially carried out to determine the chemical components of lignin recovered through water-only and dilute acid flowthrough pretreatment. Among the soluble lignin products, vanillin and syringaldehyde were found as the predominant lignin derived aromatic structures pretreated under both water-only and acid conditions (provided in Additional file 3: Figure S2 and Additional file 4: Table S2). These two compounds generally were considered derived from lignin units of coniferyl alcohol (G) and sinapyl alcohol (S). This can be speculated as derived initially from the acidic cleavage of the predominant β-O-4 bonds of lignin to phenylpropanoid structural moieties (for example, sinapaldehyde) and further oxidized to vanillin and syringaldehyde [37,38]. It was noteworthy that dilute acid conditions generated fewer phenylpropanoids than those with water only (provided in Additional file 4: Table S2). For example, no coniferyl alcohol was observed in dilute acid conditions, suggesting that 0.05% (w/w) H_2_SO_4_ with relatively lower pH was more prone to the oxidation reactions. Most of these soluble lignin compounds presented in hydrolyzates were considered as inhibitory compounds to biocatalysts in the subsequent bioconversion processes. Such hydrolyzates usually require some form of post-pretreatment detoxification to proceed effectively [39,40].

### Effects of enzyme loading and BSA addition on enzymatic hydrolysis of pretreated whole slurries

In this study, whole slurries pretreated under water-only or dilute acid conditions were hydrolyzed by enzymes at different enzyme loadings and enzymatic yields of xylose and glucose were investigated. In addition, the enzymatic hydrolysis of pretreated whole slurries with and without BSA was compared to investigate the effects of BSA treatment on digestion of both cellulosic and xylan fractions. Whole slurries pretreated under water-only conditions (that is, 270°C, 10 minutes, 25 mL/minute) and dilute acid conditions (that is, 240°C, 0.05% (w/w) H_2_SO_4_, 8 minutes, 25 mL/minute), which resulted in nearly complete biomass removal, highest total monomeric and oligomeric xylose and glucose yield, negligible sugar degradation products, as well as relatively lower liquid consumption, were applied as substrates for enzymatic hydrolysis evaluation.

As shown in Table 2, results revealed that dilute acid flowthrough pretreatment exhibited overall much better performance in enzymatic hydrolysis than water only. For example, for water-only pretreated whole slurries, about 70% enzymatic glucose yield was reached within 4 hrs at the high enzyme loading and enzymatic glucose yield gradually increased to 95% in 72 hrs. At the medium enzyme loading, only 51.5% enzymatic glucose yield was observed at 4 hrs and it improved to about 65% at 120 hrs. With the low enzyme loading, glucose yields were 41.5 and 49.1% at 4 and 120 hrs, respectively. On the contrary, dilute acid resulted in higher glucose yield of 52.7% at 4 hrs and 73.3% at 120 hrs with the lowest enzyme loading. With the medium enzyme loading, about 93% glucose yield was reached within 120 hrs. At the high enzyme loading, glucose yield was found to be >90% without BSA within 4 hrs. Results indicated that dilute acid pretreatment led to more readily digestible cellulosic derivatives. It could be explained by the fact that the recovered glucan in acid pretreatment hydrolyzate was predominately composed of glucose and soluble glucose oligomers, which totally accounted for 86.3% based on the original glucose in poplar wood. In contrast, only 52.0% yield of glucose plus soluble glucose oligomers was obtained for water-only flowthrough pretreatment, while the rest of removed cellulose (about 50%) was considered as insoluble cellulose derivatives. On the other hand, it is noteworthy that glucose yield within the initial period of 4 hrs of enzymatic hydrolysis of pretreated whole slurries with various enzyme loading was 41.5% to 91.2% for both water only and dilute acid, then glucose yield gradually increased to 49.1% to 100% at 120 hrs with prolonged hydrolysis. It indicated that when nearly complete biomass dissolution was achieved, the large portion of soluble glucose oligomers and insoluble cellulose derivatives in pretreatment hydrolyzate were quickly hydrolyzed by enzymes thus enzymatic hydrolysis of such whole slurries was more effective than hydrolysis of cellulose remained in the pretreated solid residues.

**Table 2:** Enzymatic hydrolysis of flowthrough pretreated whole slurries

**Pretreatment conditions**^**a**^	**Enzyme loading**^**b**^	**1% (w/w) Bovine serum albumin**	**Enzymatic glucose yield (%)**						**Enzymatic xylose yield (%)**					
			**t**_**1**_	**t**_**2**_	**t**_**3**_	**t**_**4**_	**t**_**5**_	**t**_**6**_	**t**_**1**_	**t**_**2**_	**t**_**3**_	**t**_**4**_	**t**_**5**_	**t**_**6**_
A	Low	Yes	45.9	48.7	51.5	51.5	53.6	54.9	43.7	79.3	88.0	89.8	90.2	91.3
A	Medium	Yes	56.0	58.5	63.6	66.3	70.8	73.0	62.8	88.1	92.8	95.0	96.0	97.8
A	High	Yes	81.6	93.1	100	100	100	100	94.9	96.8	97.0	97.8	98.0	98.3
A	Low	No	41.5	42.8	43.0	44.9	47.2	49.1	38.6	74.0	84.9	89.2	90.0	90.8
A	Medium	No	51.5	53.8	56.5	59.9	62.5	66.4	58.1	85.0	88.1	92.2	95.0	96.1
A	High	No	73.4	86.5	92.7	95.6	98.1	99.0	89.7	94.1	95.7	96.0	97.1	97.8
B	Low	Yes	57.1	72.6	75.4	77.4	77.6	79.6	48.9	82.5	90.3	91.8	92.1	92.8
B	Medium	Yes	67.3	83.1	88.3	92.8	94.7	97.7	68.3	90.6	91.0	93.2	95.5	97.1
B	High	Yes	99.5	99.8	100	100	100	100	93.7	96.7	97.8	98.1	98.2	98.8
B	Low	No	52.7	65.6	66.2	69.8	71.3	73.3	43.9	77.8	86.3	91.0	91.7	92.2
B	Medium	No	62.9	77.9	82.9	86.5	90.3	93.3	63.8	87.6	89.8	93.0	94.9	97.0
B	High	No	91.2	94.4	97.8	100	100	100	89.0	96.2	96.9	97.2	98.0	98.6

^a^A: 270°C, 10 minutes, 25 mL/minute, water only; B: 240°C, 8 minutes, 25 mL/minute, 0.05% (w/w) H_2_SO_4_. ^b^Low enzyme loading: 3 mg protein Ctec2 (2.8 filter paper units (FPU)) with 0.6 mg protein Htec2/g glucan + xylan; medium enzyme loading: 10 mg protein Ctec 2 (9.3 FPU) with 2 mg Htec2/g glucan + xylan; high enzyme loading: 100 mg protein Ctec 2 (93 FPU) with 20 mg Htec2/g glucan + xylan.

Enzymatic hydrolysis time-t_1_: 4 hrs; t_2_: 24 hrs; t_3_: 48 hrs; t_4_: 72 hrs; t_5_: 96 hrs; t_6_: 120 hrs.

Enzymatic xylose yield of pretreated whole slurries reached 94.1 and 96.8% for water only and dilute acid, respectively, within 24 hrs at the high enzyme loading (Table 2). The medium enzyme loading resulted in 92.2 and 89.2% of enzymatic xylose yield for water-only pretreated whole slurries in 72 hrs, respectively. Similar enzymatic xylose yields were found at these lower enzyme loadings for dilute acid pretreated whole slurries. Results suggested that xylooligomers in pretreated whole slurries were effectively hydrolyzed by enzymes even with low enzyme loading and that both water-only and dilute acid flowthrough pretreatment led to high yield of xylose by enzymatic hydrolysis.

It was reported that BSA treatment resulted in substantial improvement of enzymatic glucose yield from enzymatic hydrolysis of solid residues pretreated by various pretreatments [20,21]. Effects of BSA treatment on enzymatic hydrolysis of pretreated whole slurries by water only and dilute acid were investigated. Results showed that enzymatic glucose yield of water-only pretreated whole slurries with low to high enzyme loading was enhanced around 5 to 10% more than that without BSA treatment. Comparatively, the effectiveness of BSA treatment was less apparent on enzymatic hydrolysis of dilute acid pretreated whole slurries (0 to 6% enhancement) than that of water-only pretreated slurries. Slight improvement of around 0 to 5% in enzymatic xylose yield was observed with BSA addition for both water-only and dilute acid pretreated slurries. It was proposed that BSA blocked non-specific binding of cellulases, reduced inhibitory effects of pretreatment generated compounds and stabilized enzymes [41]. With most of glucan and xylan recovered in pretreatment hydrolyzate in forms of monomers, soluble oligomers and insoluble derivatives in this study, benefits of BSA treatment on improving enzymatic sugar yield were less apparent than that with pretreated solid residues in previous studies [20].

### Combined total monomer sugar yields through flowthrough pretreatment followed by enzymatic hydrolysis

The pretreated whole slurries after water-only and dilute acid flowthrough pretreatment (stage 1) subsequently underwent enzymatic hydrolysis (stage 2) to maximize mono sugar yield. Table 3 compares and summarizes the sugar yields obtained from stage 1 and stage 2 under water-only (that is, 270°C, 10 minutes, 25 mL/minute) and dilute acid (that is, 240°C, 0.05% (w/w) H_2_SO_4_, 8 minutes, 25 mL/minute) conditions that resulted in the highest total sugar yields (monomers and soluble oligomers), negligible sugar degradation products, nearly complete biomass removal and relatively lower water consumption at stage 1.

**Table 3:** **Material balance**^a^**of flowthrough pretreatment (stage 1) and enzymatic hydrolysis (stage 2)**

**Pretreatment conditions**	**Enzyme loading**^**b**^	**Stage 1**							**Stage 1 + stage 2**			
		**Soluble fractions**					**Insoluble fractions**		**Soluble fractions**			**Insoluble fractions**
		**Glu (g)**	**GOS(g)**	**Xyl (g)**	**XOS (g)**	**SL (g)**	**L-GOS (g)**	**ISL (g)**	**Glu (g)**	**Xyl (g)**	**SL (g)**	**ISL (g)**
A	High	7.5	19.7	4.0	14.7	1.2	23.8	21.8	52.7	18.6	1.2	21.8
B	Medium	31.2	15.5	6.8	12.2	0.5	4.9	23.0	50.8	18.7	0.5	23.0

^a^The calculation is based on 100 g dry weight poplar wood, which contains 54.2 g glucose, 19.1 g xylose and 23.7 g lignin; A: 270°C, water only, 25 mL/minute, 10 minutes; B: 240°C, 0.05% (w/w) H_2_SO_4_, 25 mL/minute, 8 minutes; the water consumption for water only (that is, 270°C, 10 minutes, 25 mL/minute) and dilute acid (that is, 240°C, 0.05% (w/w) H_2_SO_4_, 8 minutes, 25 mL/minute) operations was approximately 250 mL and 200 mL, respectively, which led to around 0.2 to 0.25% (w/w) overall solid to liquid ratio with a solid loading of 0.5 g. ^b^High enzyme loading: 100 mg protein Ctec 2 (93 filter paper units (FPU)) with 20 mg Htec2/g glucan + xylan; medium enzyme loading: 10 mg protein Ctec 2 (9.3 FPU) with 2 mg Htec2/g glucan + xylan. Glu, glucose; Xyl, xylose; XOS, xylooligomers; GOS, soluble glucose oligomers; L-GOS, insoluble glucan derivatives; SL, soluble lignin; ISL, insoluble lignin.

The enzyme loading employed during stage 2 for selected water-only and dilute acid pretreated slurries were high and medium enzyme loading, respectively, both of which led to >90% enzymatic glucose yield and >95% enzymatic xylose yield from corresponding samples. Results showed that on the basis of 100 g poplar wood, more than half of cellulose and nearly all xylan was converted to soluble sugars at stage 1 for the selected water-only operation: 4.0 g xylose plus 14.7 g xylooligomers, and 7.5 g glucose plus 19.7 g glucose oligomers were obtained. For dilute acid pretreatment, nearly complete polysaccharides solubilization (approximately 100% xylan and approximately 90% cellulose) led to slightly higher xylose content (6.8 g) accompanied with 12.2 g xylooligomers and much higher glucose content of 31.2 g plus 15.5 g glucose oligomers. Predominate soluble sugar fractions and insoluble sugar fractions were converted into sugar monomers at stage 2 for both selected water-only and dilute acid operations, resulting in 52.7 g glucose plus 18.6 g xylose, and 50.8 g glucose plus 18.7 g xylose, respectively. Although the material balance implied slight loss of some mass during pretreatment, the selected flowthrough conditions resulted in more than 93% glucose and 97% xylose yields after stage 1 and stage 2. Particularly, merely less than 10 filter paper units (FPU)/g glucan + xylan enzyme was required to reach over 90% total sugar (C6 and C5) yield during stage 2 for dilute acid pretreated whole slurries because around 90% cellulose was solubilized as glucose and glucose oligomers by pretreatment (stage 1).

## Conclusion

Poplar wood was pretreated through water-only and dilute acid flowthrough approaches at a temperature of 200 to 280°C and it resulted in more than 98% solid removal. Temperature was considered as the most significant factor for cellulose degradation. The cellulose removal significantly increased as temperature reached 240°C for water only and 220°C for dilute acid. Up to 100% xylan and 90% cellulose were hydrolyzed with negligible furfural and 5-HMF formation during pretreatment. Dilute acid pretreatment also resulted in higher yields of recovered xylan and cellulose as monomeric sugars in the hydrolyzate than that for water-only pretreatment. The insoluble lignin accounted for the majority of the original lignin (approximately 90%) while a small amount (approximately 15%) became soluble in the pretreated whole slurries. A larger fraction of recovered lignin was soluble with water-only pretreatment. Increasing severity enhanced total mass removal, xylan removal, lignin removal, and cellulose removal, and adding dilute sulfuric acid significantly accelerated all of the above. Dissolution of almost all biomass in hydroyzate was obtained at logR_0_ around 6.0 without acid added while a faster rate was achieved with dilute acid (logR_0_ around 5.0). Comparatively, flow rate appeared to have a less significant effect on removal of xylan, lignin, cellulose, and total mass as well as recovery yields although flow rate was associated with reaction time to affect pretreatment kinetics. Enzymatic hydrolysis of the pretreated whole slurries obtained under desired conditions for water only (270°C, 25 mL/minute, 10 minutes) and dilute acid (240°C, 0.05%(w/w) H_2_SO_4_, 25 mL/minute, 8 minutes) revealed that 93 to 97% glucose yield and 97 to 98% xylose yield were obtained. The pretreated whole slurries under selected dilute acid conditions (240°C, 0.05%(w/w) H_2_SO_4_, 25 mL/minute, 8 minutes) that resulted in much higher soluble glucose plus glucose oligomers yield (approximately 90%) at stage 1 than the water-only operation (270°C, 25 mL/minute, 10 minutes) merely required less than 10 FPU/g glucan + xylan enzyme to achieve >90% glucose yield and >95% xylose yield. The limited inhibitory compounds in the pretreated slurries showed insignificant impact on the performance of enzymes on pretreated whole slurries through BSA testing, especially for dilute acid pretreatment. In addition, the insoluble lignin was recovered from hydrolyzate with low molecular weight (<1800 Dalton). We also developed catalytic techniques to convert such technical lignin into C7- to C9-range hydrocarbons through a novel hydrodeoxygenation process in our research group [35,42,43]. Overall, both of water-only and dilute acid flowthrough pretreatments of poplar wood followed by enzymatic hydrolysis significantly enhanced monomeric sugars and lignin yields. This study proved not only high xylan and cellulose recovery due to the decrystallization of cellulose combined with solubilization of total biomass through pretreatment but also high lignin yield [10,13,15,27,44]. More importantly, the comprehensive characterization of all three major components of biomass during pretreatment under the tested conditions was reported for the first time. These findings also imply that the fundamental interactions of biomass and water and acid can be applied to understand other aqueous chemical pretreatments - their successes, pitfalls, and best optimization strategies can lend considerable insight into their sensitivity. The new insight gained will lead to obtaining of even higher yields of fermentable sugars and reactive lignin for biofuels production.

## Methods

### Feedstocks

Poplar wood provided by Forest concepts (Auburn, WA, USA) contains 48.8% cellulose, 16.8% xylan and 23.7% Klason lignin as determined by standard National Renewable Energy Laboratory Analytical Procedures (NREL LAPs) [45]. Poplar wood material was grounded with Hammermill (Hammer1067-A-1, Buffalo, NY, USA) at 4500 rpm with a 1.59-mm screen. Then the particles were collected to pass between sieve 20 mesh and sieve 40 mesh to obtain particles over a size range of 0.425 to 0.850 mm for experiments and analysis. The materials were sealed in heavy-duty zipped bags and stored at -20°C in a laboratory freezer.

### Flowthrough pretreatment

The flowthrough reactor is 1.3 cm i.d. × 15.2 cm length with an internal volume of 20.2 mL. It is constructed of 316 stainless steel parts using Vacuum Coupling Radius Seal (VCR) fittings, including one VCR male union (1.3 cm), two gasket filters (average pore size 5 μm), two VCR glands (1.3 cm × 1.3 cm), two VCR nuts, and two VCR reducing fittings (1.3 cm × 0.3 cm). All reactor parts are obtained from Swagelok Co., Richland, WA, USA. A preheating coil (0.6 cm o.d. × 0.1 cm wall, stainless steel) is connected with the reactor system and the cooling coil (0.3 cm o.d. × 0.1 cm wall). A high-pressure pump (Acuflow Series III Pumps, Fisher, Pittsburgh, PA, USA) with a flow rate range of 0 to 100 mL/minute, a pressure gauge (pressure range 0 to 1500 psi; Cole-Parmer Instrument Co., IL, USA), and a back-pressure regulator (Valve and Fitting Co., WA, USA) are used to control the flowthrough system. Biomass substrate, 0.5 g, is loaded into the reactor. Distilled water or 0.05% (w/w) sulfuric acid is pumped through the reactor to purge air and then used to pressurize the reactor to a set pressure of 225 to 1,245 psi. The reactors are heated to the target temperature (200 to 280°C) in a 4-kW fluidized sand bath (model SBL-2D, Omega engineering, Inc., CT). A thermal monitor combined with a 0.3-cm stainless steel thermocouple (Omega Engineering Co., Stamford, CT) was connected to the outlet of the flow reactor to precisely control the reaction temperature.

### Analytical methods

The pH of each liquid sample was promptly measured with a pH meter (model pH510 Series, Oakton Instruments, IL) upon cooling to room temperature. All the experiments were performed in duplicate, with the average value reported.

#### Sugar and sugar degradation products analysis

Glucose, xylose, furfural, and 5-HMF in hydrolyzates of pretreatment and enzymatic hydrolysis were analyzed using a Waters HPLC system (model 2695) equipped with a 410 refractive detector and a Waters 2695 autosampler using Waters Empower Build 1154 software (Waters Co., Milford, MA, USA). Bio-Rad Aminex HPX-87H column (Bio-Rad Laboratories, Hercules, CA, USA) was operated under 65°C. Yields of glucose, xylose, furfural, and 5-HMF were calculated as follows [46]:

(1) $$\mathrm{Glucose}\%=\frac{W_{G}\times {\mathrm{MW}}_{\mathrm{Gn}}}{W_{\mathrm{Gn}}\times {\mathrm{MW}}_{G}}\times 100\%$$

(2) $$\mathrm{Xylose}\%=\frac{W_{X}\times {\mathrm{MW}}_{\mathrm{Xn}}}{W_{\mathrm{Xn}}\times {\mathrm{MW}}_{X}}\times 100\%$$

(3) $$5‒\mathrm{HMF}\%=\frac{W_{5‒\mathrm{HMF}}\times {\mathrm{MW}}_{\mathrm{GN}}}{W_{\mathrm{Gn}}\times {\mathrm{MW}}_{5‒\mathrm{HMF}}}\times 100\%$$

(4) $$\mathrm{Furfural}\%=\frac{W_{\mathrm{Fur}}\times {\mathrm{MW}}_{\mathrm{Xn}}}{W_{\mathrm{Xn}}\times {\mathrm{MW}}_{\mathrm{Fur}}}\times 100\%$$

In these equations, W_Gn_ and W_Xn_ represent the initial weight of glucan and xylan, respectively. W_G_, W_X_, W_5-HMF_ and W_Fur_ represent the weight of glucose, xylose, 5-HMF and furfural, respectively. The unit of W consistently refers to g/100 g dw raw biomass. Molecular weight: MW_Gn_ = 162, MW_Xn_ = 132, MW_G_ = 180, MW_X_ = 150, MW_5-HMF_ = 126, MW_Fur_ = 96.

Pretreatment hydrolyzate flowing out of the flowthrough system was collected then filtered through a 0.45-μm polypropylene membrane filter (VWR, Radnor, PA, USA). The filtrate was autoclaved in 4% (w/w) sulfuric acid for 1 hr at 121°C to breakdown glucose oligomers and xylooligomers into their monomeric sugars based on standard NREL LAPs [47]. Yields of soluble glucose oligomers and xylooligomers were then calculated as follows [48]:

(5) $$\mathrm{Glucose}\;\mathrm{oligomers}\%=\frac{W_{\mathrm{TG}}‒W_{G}}{W_{\mathrm{OG}}}\times 100\%$$

(6) $$\mathrm{Xylooligomers}\%=\frac{W_{\mathrm{TX}}‒W_{X}}{W_{\mathrm{OX}}}\times 100\%$$

In these equations, W_TG_ and W_TX_ represent the total glucose and total xylose after autoclaving of filtrate; W_G_ and W_X_ represent glucose and xylose in the pretreatment filtrate before autoclaving; W_OG_ and W_OX_ represent the original glucan (as glucose) and original xylan (as xylose); The unit of W consistently refers to g/100 g dw raw biomass.

Pretreatment hydrolyzate (without filtration, not including solid residue in the reactor) was presoaked with 1% (w/w) BSA at pH 4.8 and then followed by enzymatic hydrolysis at 50°C for 168 hours with a high enzyme loading (100 mg protein Ctec 2 (93 FPU) with 20 mg Htec2/g glucan + xylan) that could guarantee maximum glucan conversion. The final glucose concentration after enzymatic hydrolysis was used to determine the total glucan recovery in pretreatment hydrolyzate. The total glucan recovery by pretreatment was calculated as follows:

(7) $$\mathrm{Total}\;\mathrm{glucan}\;\mathrm{recovery}\%=\frac{W_{\mathrm{EG}}}{W_{\mathrm{OG}}}\times 100\%$$

In this equation, W_EG_ is the total glucose after enzymatic hydrolysis; W_OG_ is the original glucan as glucose. The unit of W consistently refers to g/100 g dw raw biomass.

#### Lignin analysis

Insoluble lignin content was measured by K-lignin method [49]. Soluble lignin was estimated by UV analysis measuring absorbance at 320 nm using similar calculation of acid soluble lignin method [50]. The structure characterization of soluble lignin was determined by GC/MS analysis. The pretreated samples were filtered and extracted with dichloromethane [51], then analyzed with an Agilent gas chromatography mass spectrometer (GC, Agilent 7890A; MS, Agilent 5975C) equipped with a DB-5MS column (30 m × 320 μm × 0.25 μm). The oven temperature was programmed from 45 to 250°C at a ramping rate of 5°C/minute. Both the initial and final temperature was held for 5 minutes. The flow rate of carrier gas (helium) was 1.3 mL/minute.

### Enzymes

Commercial preparations of Novozymes Cellic® CTec2 (220 mg protein/mL, preserve 200 mg glucose/mL, 205 FPU/mL) and Novozymes Cellic® HTec2 (230 mg protein/mL, preserve 180 mg xylose/mL) were generously provided by Dr Melvin Tucker from NERL for all hydrolysis experiments. The filter paper activity of CTec2 was determined according to the standard filter paper assay [52].

### Enzymatic hydrolysis

All enzymatic hydrolysis experiments were run in duplicate under standard conditions (50°C, pH 4.8). The pretreated whole slurries (including solid residue) from flowthrough pretreatment were adjusted to the set pH with 0.1 N NaOH. A mixture of Ctec2 and Htec2 enzymes at a ratio of 5:1 based on protein weight was added at three different enzyme loadings: (1) low enzyme loading: the loadings of 3 mg protein Ctec2 (2.8 FPU) with 0.6 mg protein Htec2/g glucan + xylan; (2) medium enzyme loading: 10 mg protein Ctec 2 (9.3 FPU) with 2 mg Htec2/g glucan + xylan; and (3) high enzyme loading: 100 mg protein Ctec 2 (93 FPU) with 20 mg Htec2/g glucan + xylan, respectively. Liquid samples were taken at 4, 24, 48, 72, 96 and 120 hrs and measured directly by HPLC for monomeric sugars. In addition, BSA treatment was conducted for parts of experiments. Prior to enzyme addition to start hydrolysis, the whole pretreated slurries were presoaked with 1% (w/w) BSA 10 mg/L sodium azide for 24 hrs [20].

(8) $$\mathrm{Enzymatic}\;\mathrm{glucose}\;\mathrm{yield}\%=\frac{W_{G2}}{W_{\mathrm{TG}}‒W_{G1}}\times 100\%$$

(9) $$\mathrm{Enzymatic}\;\mathrm{xylose}\;\mathrm{yield}\%=\frac{W_{X2}}{W_{\mathrm{TX}}‒W_{X1}}\times 100\%$$

In these equations, W_G1_ and W_X1_ are the glucose and xylose released in the pretreatment; W_G2_ and W_X2_ are the glucose and xylose released in enzymatic hydrolysis; W_TG_ and W_TX_ are the total potential glucose and xylose released after enzymatic hydrolysis of whole pretreated slurries (including solid residue) with the high enzyme loading (100 mg protein Ctec 2 (93 FPU) with 20 mg Htec2/g glucan + xylan) in 168 hrs. The unit of W consistently refers to g/100 g dw raw biomass.

### Severity parameters

A severity parameter logR_0_, which is widely applied in hot water and dilute acid pretreatment [13,53,54], was used to unify our data obtained at different combinations of temperature and reaction time, which includes the preheating time.

The severity Log R_0_ is defined as follows [55]:

(10) $$\log R_{0}=\log\left[t\times \exp\left(\frac{T‒100}{14.75}\right)\right]$$

In which t is reaction time in minutes (including the preheating time); T is the hydrolysis temperature in °C, and 100°C is the reference temperature.

Because logR_0_ is the function of temperature and time as described in Equation 10, its value was calculated based on Equation 10 using the measured value of target reaction temperature from the thermal monitor and the reaction time.

## Abbreviations

5-HMF: 5-hydroxymethylfurfural; BSA: bovine serum albumin; DP: degree of polymerization; FPU: filter paper units; GC/MS: gas chromatography/mass spectrometry; HPLC: high-performance liquid chromatography.

## Competing interests

The authors declare that they have no competing interests.

## Authors’ contributions

LY and TZ carried out this study under the supervision of BY. All the authors read and accepted this final manuscript.

## Supplementary Material

Additional file 1: Figure S1 Preheating time for the target temperatures 200°C to 280°C for both water-only and 0.05% (w/w) H_2_SO_4_ flowthrough pretreatment. Click here for file

Additional file 2: Table S1 Preheating analysis for both water-only and 0.05% (w/w) H_2_SO_4_ flowthrough pretreatment. Click here for file

Additional file 3: Figure S2 Major structure of soluble lignin with water-only or 0.05% (w/w) H_2_SO_4_ flowthrough pretreatment at flow rate of 25 mL/minute within 6 minutes under **(a)** 220°C, water only ; **(b)** 240°C, water only; **(c)** 260°C, water only; **(d)** 280°C, water only; **(e)** 200°C, 0.05% (w/w) H_2_SO_4_; **(f)** 240°C, 0.05% (w/w) H_2_SO_4._ Click here for file

Additional file 4: Table S2 Major soluble aromatic compounds detected in hydrolysate by flowthrough pretreatment of poplar wood with water only and 0.05% (w/w) H_2_SO_4_. Click here for file

## References

[B1] Himmel ME Ding S Johnson DK Adney WS Nimlos MR Brady JW Foust TD Biomass recalcitrance: engineering plants and enzymes for biofuels production Science 2007 135 804 807 17289988 10.1126/science.1137016

[B2] Yang B Wyman CE Pretreatment: the key to unlocking low-cost cellulosic ethanol Biofpr 2008 2 26 40

[B3] Lynd LR Elander RT Wyman CE Likely features and costs of mature biomass ethanol technology Appl Biochem Biotechnol 1996 57–58 741 761

[B4] Tao L Aden A Elander RT Pallapolu VR Lee YY Garlock RJ Balan V Dale BE Kim Y Mosier NS Ladisch MR Falls M Holtzapple MT Sierra R Shi J Ebrik MA Redmond T Yang B Wyman CE Hames B Thomas S Warner RE Process and technoeconomic analysis of leading pretreatment technologies for lignocellulosic ethanol production using switchgrass Bioresour Technol 2011 102 11105 11114 10.1016/j.biortech.2011.07.051 21865030

[B5] Bobleter O Binder H Concin R Burtscher E Palz W, Chartier P, Hall DO The conversion of biomass to fuel raw material by hydrothermal pretreatment Energy from Biomass 1981 London: Applied Science Publishers 554 562

[B6] Lora JH Wayman M Delignification of hardwoods by auto-hydrolysis and extraction Tappi J 1978 61 47 50

[B7] Bouchard J Nguyen TS Chornet E Overend RP Analytical methodology for biomass pretreatment. part2: characterization of the filtrates and cumulative product distribution as a function of treatment severity Bioresour Technol 1991 36 121 131 10.1016/0960-8524(91)90169-K

[B8] Walsum GPV Allen SG Spencer MJ Laser MS Antal MJ Lynd LR Conversion of lignocellulosics pretreated with liquid hot water to ethanol Appl Biochem Biotech 1996 57–58 157 170

[B9] Weil J Sarikaya A Rau SL Goetz J Ladisch CM Brewer M Hendrickson R Ladisch MR Pretreatment of yellow poplar sawdust by pressure cooking in water Appl Biochem Biotech 1997 68 21 40 10.1007/BF02785978

[B10] Bobleter O Dumitriu S Hydrothermal degradation and fractionation of saccharides and polysaccharides Polysaccharides: Structural Diversity and Functional, Charpter 40 2005 2 New York: Marcel Dekker Inc 893 936

[B11] Mok WSL Antal MJ Uncatalyzed solvolysis of whole biomass hemicellulose by hot compressed liquid water Ind Eng Chem Res 1992 31 1157 1161 10.1021/ie00004a026

[B12] Vallejos ME Zambon MD Area MC da Silva Curvelo AA Low liquid–solid ratio (LSR) hot water pretreatment of sugarcane bagasse Green Chem 2012 14 1982 1989 10.1039/c2gc35397k

[B13] Yang B Wyman CE Effect of xylan and lignin removal by batch and flowthrough pretreatment on the enzymatic digestibility of corn stover cellulose Biotechnol Bioeng 2004 86 88 95 10.1002/bit.20043 15007845

[B14] Shao X Lynd L Kinetic modeling of xylan hydrolysis in co- and countercurrent liquid hot water flow-through pretreatments Bioresour Technol 2013 130 117 124 23306119 10.1016/j.biortech.2012.11.109

[B15] Phaiboonsilpa N Yamauchi K Lu X Saka S Two-step hydrolysis of Japanese cedar as treated by semi-flow hot-compressed water J Wood Sci 2010 56 331 338 10.1007/s10086-009-1099-0

[B16] Bonn G Concin R Bobleter O Hydrothermolysis-a new process for the utilization of Biomass Wood Sci Technol 1983 17 195 202 10.1007/BF00372318

[B17] Lu X Yamauchi K Phaiboonsilpa N Saka S Two-step hydrolysis of Japanese beech as treated by semi-flow hot-compressed water J Wood Sci 2009 55 367 375 10.1007/s10086-009-1040-6

[B18] Dutta A Dowe N Ibsen KN Schell DJ Aden A An economic comparison of different fermentation configurations to convert corn stover to ethanol using Z. mobilis and Saccharomyces Biotechnol Prog 2010 26 64 72 19785041 10.1002/btpr.311

[B19] Yang B Dai Z Ding S-Y Wyman CE Enzymatic hydrolysis of cellulosic biomass Biofuels 2011 2 421 450 10.4155/bfs.11.116

[B20] Yang B Wyman CE BSA treatment to enhance enzymatic hydrolysis of cellulose in lignin containing substrates Biotechnol Bioeng 2006 94 611 617 10.1002/bit.20750 16673419

[B21] Brethauer S Studer MH Yang B Wyman CE The effect of bovine serum albumin on batch and continuous enzymatic cellulose hydrolysis mixed by stirring or shaking Bioresour Technol 2011 102 6295 6298 10.1016/j.biortech.2011.02.016 21376571

[B22] Shi J Ebrik M Yang B Wyman CE The potential of cellulosic ethanol production from municipal solid waste: a technical and economic evaluation UC Energy Institute, Energy development and technology, Volume 015 2009 CA: UC Energy Institute

[B23] Stuhler SL Wyman CE Estimation of temperature transients for biomass pretreatment in tubular batch reactors and impact on xylan hydrolysis kinetics Appl Biochem Biotech 2003 105 101 114 10.1385/ABAB:105:1-3:101 12721478

[B24] Yang B Wyman CE Characterization of the degree of polymerization of xylooligomers produced by flowthrough hydrolysis of pure xylan and corn stover with water Bioresour Technol 2008 99 5756 5762 10.1016/j.biortech.2007.10.054 18096381

[B25] Ragauskas AJ Williams CK Davison BH Britovsek G Cairney J Eckert CA Frederick WJ Hallett JP Leak DJ Liotta CL Mielenz JR Murphy R Templer R Tschaplinski T The path forward for biofuels and biomaterials Science 2006 311 484 489 10.1126/science.1114736 16439654

[B26] Wada M Sugiyama J Okano T Native celluloses on the basis of two crystalline phase (Iα/Iβ) system J Appl Polym Sci 1993 49 1491 1496 10.1002/app.1993.070490817

[B27] Liu C Wyman CE The effect of flow rate of compressed hot water on xylan, lignin, and total mass removal from corn stover Ind Eng Chem Res 2003 42 5409 5416 10.1021/ie030458k

[B28] Xiang Q Lee YY Pettersson PO Torget R Heterogeneous aspects of acid hydrolysis of α-cellulose Appl Biochem Biotech 2003 105 505 514 10.1385/abab:107:1-3:505 12721431

[B29] Xiang Q Lee YY Torget RW Kinetics of glucose decomposition during dilute-acid hydrolysis of lignocellulosic biomass Appl Biochem Biotech 2004 113–116 1127 1138 10.1385/abab:115:1-3:1127 15054258

[B30] Rosatella AA Simeonov SP Frade RFM Afonso CAM 5-Hydroxymethylfurfural (HMF) as a building block platform: biological properties, synthesis and synthetic applications Green Chem 2011 13 754 793 10.1039/c0gc00401d

[B31] Marcotullio G De JW Chloride ions enhance furfural formation from D-xylose in dilute aqueous acidic solutions Green Chem 2010 12 1739 1746 10.1039/b927424c

[B32] Song J Fan H Ma J Han B Conversion of glucose and cellulose into value-added products in water and ionic liquids Green Chem 2013 15 2619 2635 10.1039/c3gc41141a

[B33] Qian X Nimlos MR Davis M Johnson DK Himmel ME Ab initio molecular dynamics simulations of β-D-glucose and β-D-xylose degradation mechanisms in acidic aqueous solution Carbohydr Res 2005 340 2319 2327 10.1016/j.carres.2005.07.021 16095579

[B34] Zeng JJ Tong ZH Wang LT Zhu JY Ingram L Isolation and structural characterization of sugarcane bagasse lignin after dilute phosphoric acid plus steam explosion pretreatment and its effect on cellulose hydrolysis Bioresour Technol 2014 154 274 281 24412855 10.1016/j.biortech.2013.12.072

[B35] Laskar DD Yang B Wang H Lee J Pathways for biomass-derived lignin to hydrocarbon fuels Biofuels Bioprod Bior 2013 7 602 626 10.1002/bbb.1422

[B36] Trajano HL Engle NL Foston M Ragauskas AJ Tschaplinski TJ Wyman CE The fate of lignin during hydrothermal pretreatment Biotechnol Biofuels 2013 6 110 125 10.1186/1754-6834-6-110 23902789 PMC3751430

[B37] Laskar DD Zeng J Yan L Chen S Yang B Characterization of lignin derived from water-only flowthrough pretreatment of Miscanthus Ind Crop Prod 2013 50 391 399

[B38] Zhuang X Yu Q Wang W Qi W Wang Q Tan X Yuan Z Decomposition behavior of hemicellulose and lignin in the step-change flow rate liquid hot water Appl Biochem Biotech 2012 168 206 218 10.1007/s12010-011-9468-8 22270547

[B39] Kim Y Ximenes E Mosier NS Ladisch MR Soluble inhibitors/deactivators of cellulase enzymes from lignocellulosic biomass Enzyme Microb Technol 2011 48 408 415 10.1016/j.enzmictec.2011.01.007 22112958

[B40] Joensson LJ Alriksson B Nilvebrant NO Bioconversion of lignocellulose: inhibitors and detoxification Biotechnol Biofuels 2013 6 16 25 10.1186/1754-6834-6-16 23356676 PMC3574029

[B41] Yang B Wyman C Lignin blockers and uses thereof US Patent 2013 8580541 B2

[B42] Laskar DD Tucker M Chen X Helms G Yang B Noble-metal catalyzed hydrodeoxygenation of biomass-derived lignin to aromatic hydrocarbons Green Chem 2014 16 897 910 10.1039/c3gc42041h

[B43] Yang B Laskar DD Apparatus and process for preparing reactive lignin with high yield from plant biomass for production of fuels and chemicals Patent Cooperation Treaty 2014 PCT/US2013/038927 in press

[B44] Bobleter O Binder H Dynamic hydrothermal degradation of wood Holzforschung 1980 34 48 51 10.1515/hfsg.1980.34.2.48

[B45] Sluiter A Hames B Ruiz R Scarlata C Sluiter J Templeton D Crocker D Determination of structural carbohydrates and lignin in biomass 2008 Golden, CO: National Renewable Energy Laboratory Analytical Procedure

[B46] Yan L Laskar DD Lee S-J Yang B Aqueous phase catalytic conversion of agarose to 5-hydroxymethylfurfural by metal chlorides RSC Adv 2013 3 24090 24098 10.1039/c3ra43293a

[B47] Sluiter A Hames B Ruiz R Scarlata C Sluiter J Templeton D Determination of sugars, byproducts, and degradation products in liquid fraction process samples 2006 Golden, CO: National Renewable Energy Laboratory Analytical Procedure

[B48] Zhang T Wyman CE Jakob K Yang B Rapid selection and identification of miscanthus genotypes with enhanced glucan and xylan yields from hydrothermal pretreatment followed by enzymatic hydrolysis Biotechnol Biofuels 2012 5 56 69 10.1186/1754-6834-5-56 22863302 PMC3494522

[B49] Templeton D Ehrman T Determination of acid-insoluble lignin in biomass 1995 Golden, CO: National Renewable Energy Laboratory Analytical Procedure

[B50] Ehrman T Determination of acid-soluble lignin in biomass 1996 Golden, CO: National Renewable Energy Laboratory Analytical Procedure

[B51] Caldeira I Clímaco MC de Sousa RB Belchior AP Volatile composition of oak and chestnut woods used in brandy ageing: modification induced by heat treatment J Food Eng 2006 76 202 211 10.1016/j.jfoodeng.2005.05.008

[B52] Adney B Baker J Measurement of cellulase activities 2008 Golden, CO: National Renewable Energy Laboratory Analytical Procedure

[B53] Yang B Tucker M Wyman CE Laboratory pretreatment systems to understand biomass deconstruction Aqueous Pretreatment of Plant Biomass for Biological and Chemical Conversion to Fuels and Chemicals 2013 West Sussex: John Wiley&Sons, Ltd 489 514

[B54] Lloyd TA Wyman CE Combined sugar yields for dilute sulfuric acid pretreatment of corn stover followed by enzymatic hydrolysis of the remaining solids Bioresour Technol 2005 96 1967 1977 10.1016/j.biortech.2005.01.011 16112484

[B55] Overend RP Chornet E Fractionation of lignocellulosics by steam aqueous pretreatments Phil Trans R Soc Lond 1987 321 523 536 10.1098/rsta.1987.0029

