# Supplementary material for: Enhancement of total sugar and lignin yields through dissolution of poplar wood by hot water and dilute acid flowthrough pretreatment
Source: Biotechnol Biofuels. 2014 May 23;7:76. doi: 10.1186/1754-6834-7-76 (PMC4040120; doi:10.1186/1754-6834-7-76)

**Figure S1. Preheating time for the target temperatures 200^o^C－280^o^C for both water-only and 0.05%(w/w) H_2_SO_4_ flowthrough pretreatment.**


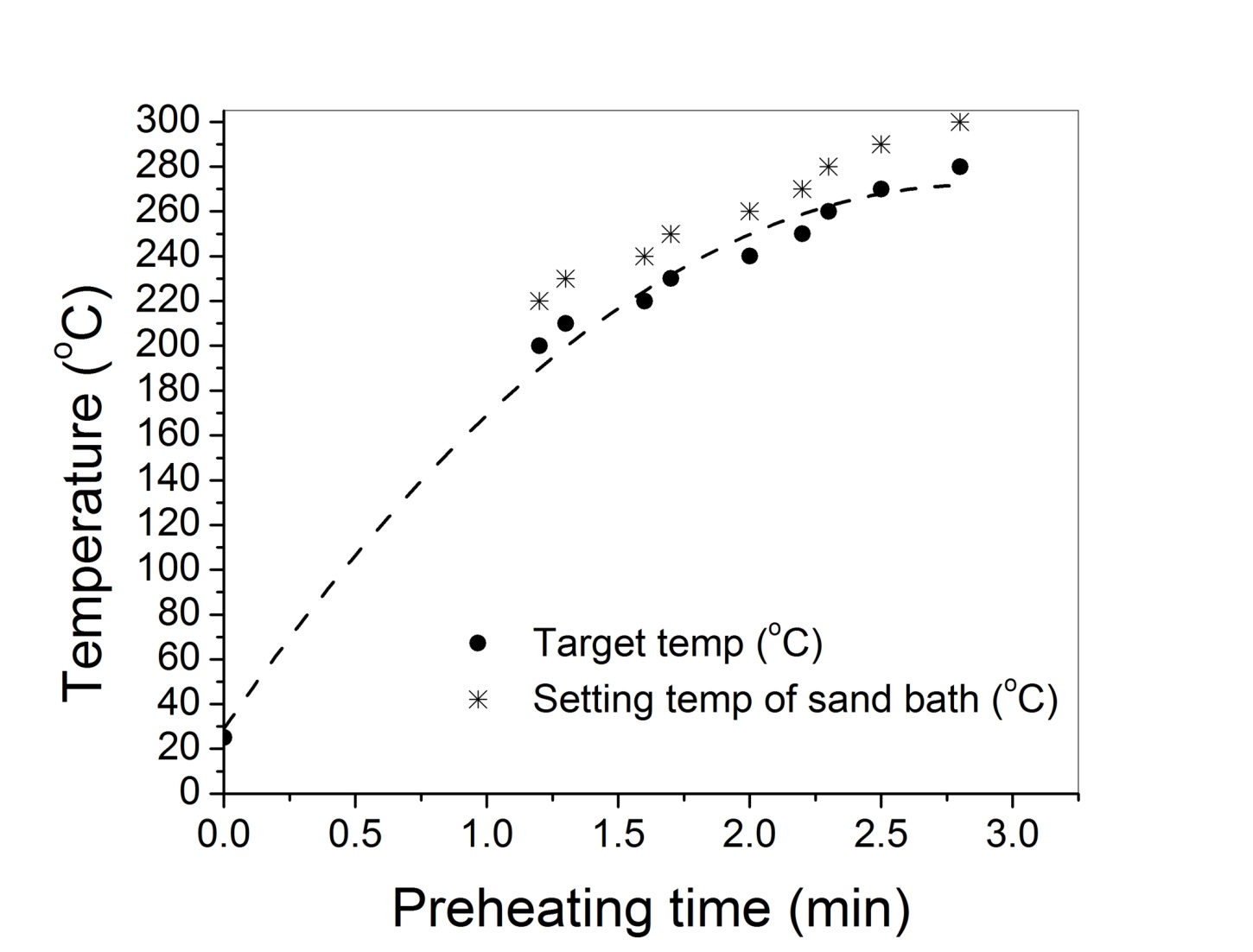

Supplement: Additional file 1: Figure S1 — Preheating time for the target temperatures 200°C to 280°C for both water-only and 0.05% (w/w) H2SO4 flowthrough pretreatment. [file 1754-6834-7-76-S1.docx]
