# Supplementary material for: Enhancement of total sugar and lignin yields through dissolution of poplar wood by hot water and dilute acid flowthrough pretreatment
Source: Biotechnol Biofuels. 2014 May 23;7:76. doi: 10.1186/1754-6834-7-76 (PMC4040120; doi:10.1186/1754-6834-7-76)
Supplement: Additional file 2: Table S1 — Preheating analysis for both water-only and 0.05% (w/w) H2SO4 flowthrough pretreatment. [file 1754-6834-7-76-S2.docx]

**Table S1.** Preheating analysis for both water-only and 0.05% (w/w) H_2_SO_4_ flowthrough pretreatment.

| Conditions | Xylan removal (%) | Lignin removal (%) | Cellulose removal (%) | Yield in pretreatment hydrolyzates (%) | | | | | | |
| --- | --- | --- | --- | --- | --- | --- | --- | --- | --- | --- |
|  |  |  |  | xylose | xylooligomers | Glucose | Glucose oligomers | furfural | 5-HMF | Soluble lignin |
| A1a | 76.2 | 52.1 | 2.6 | 1.5 | 73.2 | 0 | 0.8 | 0 | 0 | 3.8 |
| A1b | 80.3 | 56.6 | 3.5 | 3.2 | 75.5 | 0 | 1.3 | 0 | 0 | 2.6 |
| A1c | 84.0 | 59.2 | 4.9 | 0 | 83.5 | 0 | 1.5 | 0 | 0 | 0 |
| A2a | 78.5 | 55.3 | 7.5 | 3.1 | 72.6 | 0.7 | 3.1 | 0 | 0 | 4.5 |
| A2b | 81.3 | 57.6 | 10.2 | 2.0 | 78.5 | 0.2 | 3.6 | 0 | 0 | 3.0 |
| A2c | 88.1 | 64.2 | 12.0 | 1.8 | 86.1 | 0 | 3.9 | 0 | 0 | 0.6 |
| A3a | 83.5 | 62.6 | 23.9 | 7.7 | 73.5 | 3.0 | 6.8 | 0 | 0 | 4.9 |
| A3b | 87.3 | 65.4 | 28.7 | 6.5 | 79.7 | 2.5 | 10.8 | 0 | 0 | 3.3 |
| A3c | 93.2 | 72.3 | 33.1 | 6.0 | 87.1 | 0 | 12.3 | 0 | 0 | 1.2 |
| A4a | 85.3 | 66.7 | 30.1 | 10.3 | 73.5 | 4.7 | 7.2 | 0 | 0 | 6.3 |
| A4b | 90.2 | 68.1 | 33.3 | 9.2 | 79.5 | 3.8 | 13.2 | 0 | 0 | 5.1 |
| A4c | 95.0 | 76.2 | 37.1 | 8.9 | 86.8 | 0 | 19.8 | 0 | 0 | 2.7 |
| A5a | 87.3 | 69.5 | 34.7 | 15.8 | 69.5 | 7.2 | 10.8 | 0 | 0 | 7.6 |
| A5b | 92.6 | 73.0 | 37.5 | 13.7 | 78.2 | 5.5 | 13.9 | 0 | 0 | 5.3 |
| A5c | 97.1 | 79.1 | 40.9 | 13.0 | 85.1 | 2.3 | 22.8 | 0 | 0 | 3.6 |
| A6a | 97.3 | 73.6 | 36.8 | 18.3 | 77.7 | 10.3 | 12.8 | 0 | 0 | 7.3 |
| A6b | 99.2 | 77.0 | 40.7 | 16.1 | 80.2 | 8.9 | 15.2 | 0 | 0 | 4.7 |
| A6c | 100 | 82.2 | 43.1 | 14.3 | 85.6 | 3.5 | 23.2 | 0 | 0 | 3.1 |
| A7a | 100 | 75.1 | 39.8 | 22.9 | 74.7 | 14.7 | 18.3 | 0.6 | 1.2 | 7.1 |
| A7b | 100 | 79.5 | 42.3 | 19.3 | 79.5 | 12.0 | 21.7 | 0 | 0 | 3.5 |
| A7c | 100 | 83.6 | 45.0 | 14.7 | 84.7 | 4.2 | 32.5 | 0 | 0 | 2.3 |
| B1a | 71.6 | 50.2 | 2.2 | 16.5 | 55.3 | 0 | 1.3 | 0 | 0 | 1.1 |
| B1b | 72.8 | 52.3 | 3.7 | 12.5 | 60.1 | 0 | 2.9 | 0 | 0 | 0.8 |
| B1c | 75.2 | 53.2 | 5.5 | 5.3 | 69.2 | 0 | 3.6 | 0 | 0 | 0 |
| B2a | 80.8 | 61.5 | 9.8 | 21.6 | 58.7 | 3.2 | 4.3 | 0 | 0 | 2.5 |
| B2b | 82.3 | 63.5 | 10.3 | 15.7 | 65.8 | 1.2 | 6.6 | 0 | 0 | 1.3 |
| B2c | 84.4 | 66.3 | 12.5 | 6.2 | 75.2 | 0 | 7.2 | 0 | 0 | 0.4 |
| B3a | 85.3 | 66.2 | 34.2 | 19.8 | 63.9 | 18.2 | 11.7 | 0 | 0 | 2.8 |
| B3b | 88.7 | 70.8 | 37.8 | 13.3 | 70.8 | 11.6 | 20.1 | 0 | 0 | 2.0 |
| B3c | 92.3 | 75.3 | 39.2 | 9.8 | 80.5 | 4.2 | 27.5 | 0 | 0 | 0.9 |
| B4a | 89.3 | 71.5 | 38.5 | 25.2 | 63.2 | 20.6 | 16.3 | 0 | 0 | 3.2 |
| B4b | 92.6 | 75.2 | 40.2 | 17.8 | 73.9 | 13.8 | 23.5 | 0 | 0 | 2.5 |
| B4c | 95.3 | 79.3 | 43.5 | 11.5 | 83.2 | 9.3 | 26.7 | 0 | 0 | 1.3 |
| B5a | 92.2 | 75.5 | 40.5 | 30.6 | 61.2 | 22.3 | 17.2 | 0 | 0 | 2.6 |
| B5b | 95.3 | 79.5 | 45.1 | 28.3 | 66.1 | 19.8 | 21.9 | 0 | 0 | 1.8 |
| B5c | 97.2 | 83.6 | 47.8 | 18.7 | 77.0 | 13.6 | 29.7 | 0 | 0 | 0.8 |
| B6a | 98.3 | 80.6 | 42.5 | 35.6 | 61.3 | 24.7 | 15.2 | 0 | 0 | 2.1 |
| B6b | 100 | 84.3 | 46.5 | 30.3 | 68.8 | 22.6 | 22.3 | 0 | 0 | 1.2 |
| B6c | 100 | 87.7 | 49.2 | 22.8 | 75.1 | 17.5 | 27.8 | 0 | 0 | 0.5 |
|  |  |  |  |  |  |  |  |  |  |  |

| Water-only conditions | | |  | 0.05%(w/w) H_2_SO_4_ conditions | | |
| --- | --- | --- | --- | --- | --- | --- |
| Sample name | Target temperature (^o^C) | Flow rate (mL/min) |  | Sample name | Target temperature (^o^C) | Flow rate (mL/min) |
| A1a | 220 | 10 |  | B1a | 200 | 10 |
| A1b | 220 | 25 |  | B1b | 200 | 25 |
| A1c | 220 | 62.5 |  | B1c | 200 | 62.5 |
| A2a | 230 | 10 |  | B2a | 210 | 10 |
| A2b | 230 | 25 |  | B2b | 210 | 25 |
| A2c | 230 | 62.5 |  | B2c | 210 | 62.5 |
| A3a | 240 | 10 |  | B3a | 220 | 10 |
| A3b | 240 | 25 |  | B3b | 220 | 25 |
| A3c | 240 | 62.5 |  | B3c | 220 | 62.5 |
| A4a | 250 | 10 |  | B4a | 230 | 10 |
| A4b | 250 | 25 |  | B4b | 230 | 25 |
| A4c | 250 | 62.5 |  | B4c | 230 | 62.5 |
| A5a | 260 | 10 |  | B5a | 240 | 10 |
| A5b | 260 | 25 |  | B5b | 240 | 25 |
| A5c | 260 | 62.5 |  | B5c | 240 | 62.5 |
| A6a | 270 | 10 |  | B6a | 250 | 10 |
| A6b | 270 | 25 |  | B6b | 250 | 25 |
| A6c | 270 | 62.5 |  | B6c | 250 | 62.5 |
| A7a | 280 | 10 |  |  |  |  |
| A7b | 280 | 25 |  |  |  |  |
| A7c | 280 | 62.5 |  |  |  |  |
