# Supplementary figures and images for: Enhancement of total sugar and lignin yields through dissolution of poplar wood by hot water and dilute acid flowthrough pretreatment
Source: Biotechnol Biofuels. 2014 May 23;7:76. doi: 10.1186/1754-6834-7-76 (PMC4040120; doi:10.1186/1754-6834-7-76)

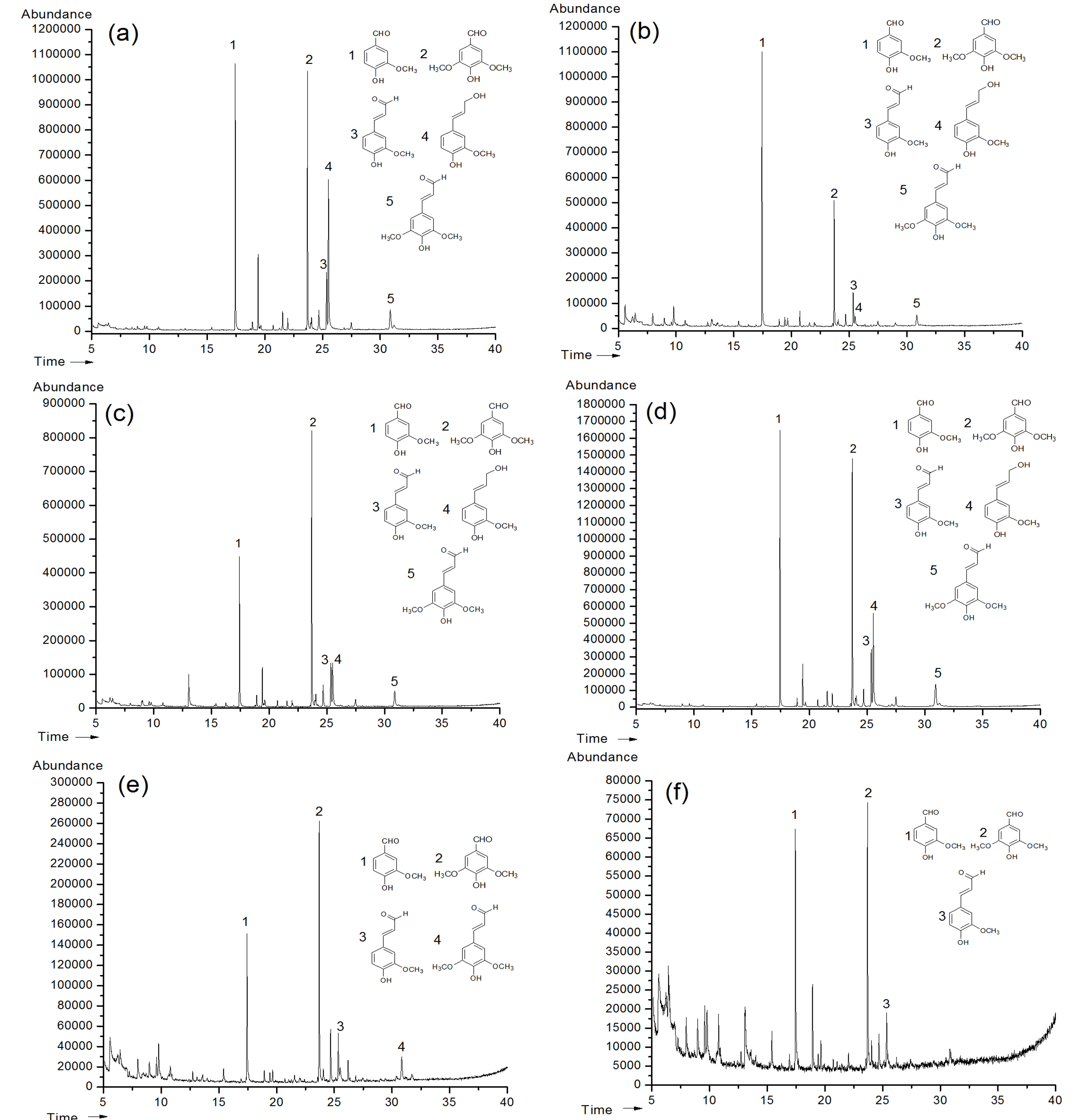

Supplement: Additional file 3: Figure S2 — Major structure of soluble lignin with water-only or 0.05% (w/w) H2SO4 flowthrough pretreatment at flow rate of 25 mL/minute within 6 minutes under (a) 220°C, water only ; (b) 240°C, water only; (c) 260°C, water only; (d) 280°C, water only; (e) 200°C, 0.05% (w/w) H2SO4; (f) 240°C, 0.05% (w/w) H2SO4. [file 1754-6834-7-76-S3.jpeg]
