# Supplementary material for: Enhancement of total sugar and lignin yields through dissolution of poplar wood by hot water and dilute acid flowthrough pretreatment
Source: Biotechnol Biofuels. 2014 May 23;7:76. doi: 10.1186/1754-6834-7-76 (PMC4040120; doi:10.1186/1754-6834-7-76)
Supplement: Additional file 4: Table S2 — Major soluble aromatic compounds detected in hydrolysate by flowthrough pretreatment of poplar wood with water only and 0.05% (w/w) H2SO4. [file 1754-6834-7-76-S4.docx]

**Table S2. Major soluble aromatic compounds detected in hydrolysate by flowthrough pretreatment of poplar wood with water-only and 0.05% (w/w) H_2_SO_4_.** + 0－10%; ++ 10%－20%; +++ 20%－50%

| Aromatic compounds | Relative abundance | | | | | | Structure | MW |
| --- | --- | --- | --- | --- | --- | --- | --- | --- |
|  | 220^o^C  HW^a^ | 240^o^C  HW^a^ | 260^o^C  HW^a^ | 280^o^C  HW^a^ | 200^o^C  Acid^b^ | 240^o^C  Acid^b^ |  |  |
| Vanillin | +++ | +++ | +++ | +++ | +++ | +++ |  | 152 |
| Syringaldehyde | +++ | +++ | +++ | +++ | +++ | +++ |  | 182 |
| Coniferaldehyde | + | + | + | + | + | + |  | 178 |
| Coniferyl alcohol | ++ | + | + | + | － | － |  | 180 |
| Sinapaldehyde | + | + | + | + | + | － |  | 208 |

a: Water-only, 25mL/min, 6min

b: 0.05%(w/w) H_2_SO_4_, 25mL/min, 6min
